# Supplementary material for: Causal Relationship between Aging and Anorexia Nervosa: A White-Matter-Microstructure-Mediated Mendelian Randomization Analysis
Source: Biomedicines. 2024 Aug 16;12(8):1874. doi: 10.3390/biomedicines12081874 (PMC11351342; doi:10.3390/biomedicines12081874)
Supplement: Supplementary file 1 [file biomedicines-12-01874-s001.zip › Supplementary Figure/Figure S7/Single SNP plot of MR analysis for ubm-b-1923 to AN.pdf]

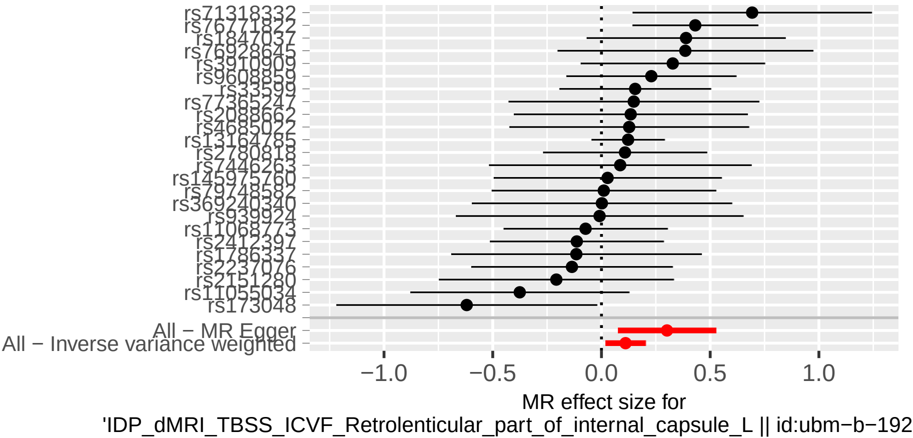
rs71318332
rs76771822
rs1847037
rs76928645
rs3910909
rs9608859
rs33599
rs77365247
rs2088662
rs4685022
rs13164785
rs2780818
rs7446263
rs145975760
rs79748582
rs369240340
rs939924
rs11068773
rs2412397
rs1786337
rs2237076
rs2151280
rs11055034
rs173048
All − MR Egger
All − Inverse variance weighted
−1.0
−0.5
0.0
0.5
1.0
MR effect size for
'IDP_dMRI_TBSS_ICVF_Retrolenticular_part_of_internal_capsule_L || id:ubm−b−192
